# Devitalizing Pulps

**Published:** 1888-05

**Authors:** 


### DEVITALIZING PULPS.

There are few things in dentistry that are so often unskillfully done as the application of arsenical paste for the devitalization of pulps. We believe that much of the pain of which so many operators complain as attendant upon this process, is due to the wretched manner in which the agent is too often used.

In the first place, perhaps the rubber-dam is not applied, but the saliva has almost unchecked entrance into the cavity and the paste is not left in proper position, even if it be not altogether washed out. All manner of debris is left in the cavity to obstruct the rapid action of the agent, and to cause an unnecessary irritation. The paste is not applied directly to the pulp, but is placed almost anywhere, so that it will be in contact with the tooth, and lastly, it is, perhaps, covered with cotton dipped in that abomination—a sandarach solution. What wonder that the results are unsatisfactory.

For the proper devitalization of a live pulp it is essential that the agent be placed in immediate contact with the pulp. All debris and loose particles should be carefully removed, and this can best be done—in part at least—by thoroughly rinsing the cavity with warm water.

Excavation is not essential, but the taking out of all debris is. The rubber dam should be applied and the cavity dried as perfectly as possible. Then, the pulp being exposed completely at some point, a minute particle of the paste should be directly applied, covered with a small cap cut from thin rolled tin or some other metal, and made concave by pressure with the rounded end of an excavator, and the cavity carefully sealed with wax, gutta-percha, or some impermeable covering. We have found modeling compound an excellent material when warmed. If this course is followed, pain will be the exception and not the rule, and should it unfortunately succeed it will be very fleeting in its character.

When the pulp exposure is from attrition or fracture, and there is no cavity of retention, a small concave cap may be made of wax, the paste placed in it, and the tooth being carefully dried and the wax warmed, it may be made to adhere sufficiently long for devitalization.

If any one really wishes to raise a toothache that shall cause him to be remembered, let him put arsenical paste in a wet cavity, the pulp being covered with refuse matter and decayed dentine, and then let him cap the climax of the outrage by thrusting into the outer cavity cotton wet with a sandarach solution. This will permeate the whole cavity, encapsule the arsenical paste and prevent its action, while it serves as a constant irritant. In a few hours it will decompose, and the cavity will become foul almost beyond conception. We think it is really the worst covering for a temporary dressing of which we have any knowledge, and we have had experience enough with it to be an expert in judging its demerits.

Does any one think that the method here recommended is too troublesome? Well, if his practice of dentistry rests upon a desire to avoid painstaking and labor, our advice to him is to drop it and seek some less irksome occupation.

